# Involvement of Oxidative Stress in Suppression of Insulin Biosynthesis under Diabetic Conditions

**DOI:** 10.3390/ijms131013680

**Published:** 2012-10-22

**Authors:** Hideaki Kaneto, Taka-aki Matsuoka

**Affiliations:** Department of Metabolic Medicine, Osaka University Graduate School of Medicine, 2-2 Yamadaoka, Suita, Osaka 565-0871, Japan; E-Mail: matsuoka@endmet.med.osaka-u.ac.jp

**Keywords:** type 2 diabetes, pancreatic β-cells, glucose toxicity

## Abstract

Type 2 diabetes is characterized by pancreatic β-cell dysfunction and insulin resistance, and the number of patients has markedly increased worldwide. In the diabetic state, hyperglycemia *per se* and subsequent induction of oxidative stress decrease insulin biosynthesis and secretion, leading to the aggravation of Type 2 diabetes. In addition, there is substantial reduction in expression and/or activities of several insulin gene transcription factors. This process is known as β-cell glucose toxicity, which is often observed under diabetic conditions. Taken together, it is likely that oxidative stress explains, at least in part, the molecular mechanism for β-cell glucose toxicity, which is often observed in Type 2 diabetes.

## 1. Natural History of Pancreatic β-Cell Failure Which Is Often Observed in Type 2 Diabetes

Type 2 diabetes is characterized by pancreatic β-cell dysfunction and insulin resistance. First, overeating and/or obesity lead to the development of insulin resistance, and normal β-cells secrete a larger amount of insulin to compensate for the increased insulin resistance. Next, large adipocytes secrete more free fatty acids (FFAs) and/or various inflammatory cytokines, which gradually deteriorate β-cell function and finally lead to the onset of diabetes. This process is known as “β-cell lipotoxicity”. Indeed, it has been reported that when islets or β-cell-derived cell lines are exposed to FFAs, oxidative stress is induced, which leads to the reduction of insulin secretion [1–5]. It has been also reported that FFA-mediated induction of inducible nitric oxide synthase (iNOS) and excess nitric oxide (NO) generation are involved in the progression of β-cell dysfunction [6]. It is noted here that since most of these experiments were performed in a cell culture system, further *in vivo* studies would be necessary to demonstrate the importance of “β-cell lipotoxicity” in the deterioration of β-cell function. In addition, the primary cause of Type 2 diabetes is not necessarily due to the aggravation of insulin resistance induced by overeating and/or obesity. It has been thought recently that Type 2 diabetes is fundamentally a polygenic disease involving primary β-cell insufficiency. Indeed, obesity and insulin resistance are not necessarily observed in Type 2 diabetes.

Once hyperglycemia becomes apparent, β-cell function such as insulin biosynthesis and secretion progressively deteriorates. This process is known as “β-cell glucose toxicity” which is often observed under diabetic conditions. In the diabetic state, hyperglycemia *per se* and subsequent induction of oxidative stress decrease insulin gene expression and secretion and finally bring about apoptotic cell death [7–32]. It is noted that although a variety of factors including ER stress, inflammatory cytokines and amyloid fibrils are likely involved in the mechanism for glucose toxicity which includes β-cell dysfunction and β-cell death, in this review we focus on the role of oxidative stress in the progression of β-cell dysfunction, especially suppression of insulin biosynthesis, found in Type 2 diabetes.

Furthermore, very recently a new concept was proposed about the natural history of β-cell failure; it was reported that loss of β-cell mass was due to β-cell dedifferentiation, but not due to β-cell death. Indeed, the lineage-tracing experiments showed that dedifferentiated β-cells converted into progenitor-like cells and adopted the α-cell fate. These findings show that the process of dedifferentiation of β-cells plays a crucial role in the natural history of β-cell failure [33].

## 2. Oxidative Stress Is involved in Pancreatic β-Cell Glucose Toxicity, Which Is Often Observed in Type 2 Diabetes

**Figure 1:**
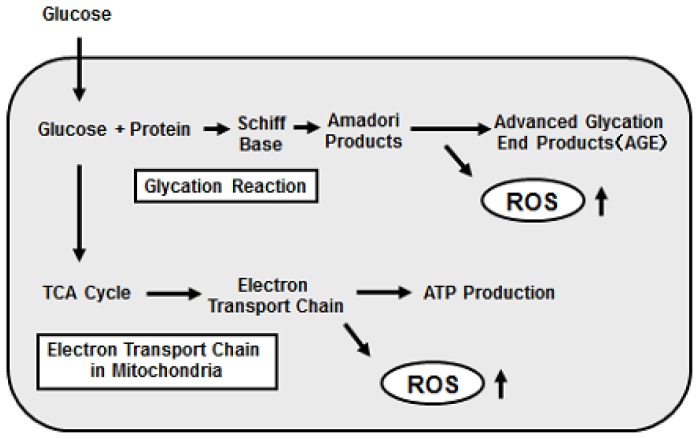
Production of reactive oxygen species (ROS) under diabetic conditions. ROS are produced by various pathways under diabetic conditions and are involved in the deterioration of pancreatic β-cell function. Hyperglycemia induces ROS through activation of the glycation reaction and electron transport chain in mitochondria.

It has been shown that oxidative stress is provoked in various tissues under diabetic conditions. There are several sources of reactive oxygen species (ROS) in cells such as the non-enzymatic glycosylation reaction [34], the electron transport chain in mitochondria [35]. The glycation reaction produces Schiff base, Amadori product, and finally advanced glycosylation end products (AGE). During the process, oxidative stress is provoked. The electron transport chain in mitochondria is also an important pathway to induce oxidative stress. Under diabetic conditions, the electron transport chain is activated, which leads to production of larger amounts of ROS (Figure 1).

Under diabetic conditions, oxidative stress is induced and involved in the β-cell glucose toxicity. β-Cells express GLUT2, a high-Km glucose transporter, and thereby display highly efficient glucose uptake when exposed to a high glucose concentration. Indeed, it was shown that expression levels of oxidative stress markers such as 8-hydroxy-2′-deoxyguanosine (8-OHdG) and 4-hydroxy-2,3-nonenal (4-HNE) were increased in islets under diabetic conditions. In addition, β-cells are rather vulnerable to oxidative stress due to the relatively low expression of antioxidant enzymes such as catalase and glutathione peroxidase [36,37]. Therefore, it is likely that oxidative stress is involved in the deterioration of β-cell function found in diabetes. It was shown that when β-cell-derived cell lines or rat-isolated islets were exposed to oxidative stress, insulin gene promoter activity and mRNA expression were suppressed. In addition, when they were exposed to oxidative stress, bindings of PDX-1 and/or MafA to the insulin gene promoter were reduced. It is noted here that PDX-1 plays a crucial role in pancreas development, β-cell differentiation, induction of surrogate β-cells, and maintenance of mature β-cell function [38–50] and that MafA is a recently isolated β-cell-specific transcription factor and functions as a potent activator of insulin gene transcription [51–56].

**Figure 2:**
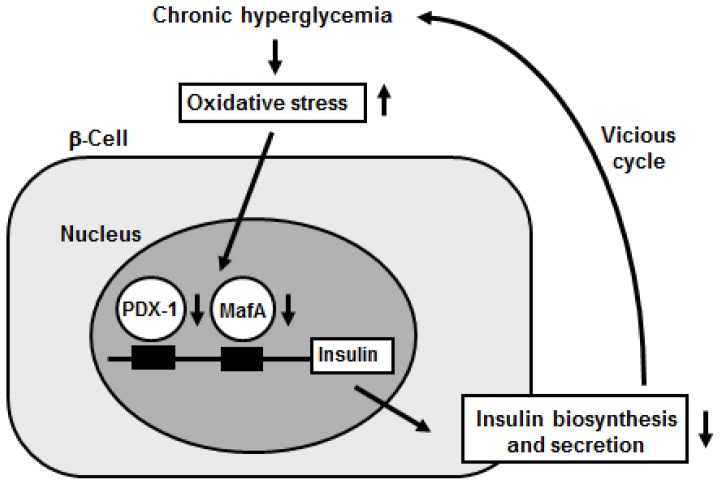
Involvement of oxidative stress in pancreatic β-cell glucose toxicity in Type 2 diabetes. Hyperglycemia and subsequent induction of oxidative stress suppress nuclear expression of pancreatic transcription factors PDX-1 and MafA, which leads to suppression of insulin biosynthesis and secretion. Therefore, it is likely that induction of oxidative stress and suppression of PDX-1 and MafA are involved in β-cell glucose toxicity found in Type 2 diabetes.

Furthermore, it was shown that the decrease of insulin gene expression after chronic exposure to a high glucose concentration was prevented by treatment with antioxidants [19,26,29–32]. Reduction of expression and/or DNA binding activities of PDX-1 and MafA by chronic exposure to high glucose was also prevented by an antioxidant treatment. These results suggest that chronic hyperglycemia suppresses insulin biosynthesis and secretion by increasing oxidative stress, accompanied by reduction of expression and/or DNA binding activities of two important pancreatic transcription factors PDX-1 and MafA. Therefore, it is likely that the alteration of such transcription factors explains, at least in part, the suppression of insulin biosynthesis and secretion, and thereby is involved in β-cell glucose toxicity (Figure 2). Indeed, it was shown that the antioxidant treatment retained glucose-stimulated insulin secretion and moderately ameliorated glucose tolerance in obese diabetic C57BL/KsJ-db/db mice [26]. Beta-Cell mass was significantly larger in the mice treated with the antioxidants, and insulin content was preserved by the antioxidant treatment. Furthermore, PDX-1 expression was more clearly visible in the nuclei of β-cells after the antioxidant treatment [26]. Similar effects were observed with Zucker diabetic fatty rats, another model animal for Type 2 diabetes [19]. Therefore, it is likely that antioxidant treatment can protect β-cells against glucose toxicity. It is noted, however, that since it remains unclear at this point whether the antioxidant treatment can have positive effects on insulin gene expression even in the presence of continuing hyperglycemia, further studies would be necessary to clarify this point.

## 3. Activation of the JNK Pathway Is Involved in Pancreatic β-Cell Glucose Toxicity

It has been suggested that activation of the c-Jun N-terminal kinase (JNK) pathway is involved in pancreatic β-cell dysfunction found in Type 2 diabetes. It was reported that activation of the JNK pathway is involved in reduction of insulin gene expression by oxidative stress and that suppression of the JNK pathway can protect β-cells from oxidative stress [57]. When isolated rat islets were exposed to oxidative stress, the JNK pathway was activated, preceding the decrease of insulin gene expression. Adenoviral overexpression of dominant-negative type JNK1 (DN-JNK) protected insulin gene expression and secretion from oxidative stress. These results were correlated with change in the binding of PDX-1 to insulin gene promoter. Adenoviral overexpression of DN-JNK preserved PDX-1 DNA binding activity in the face of oxidative stress, while WT-JNK overexpression decreased PDX-1 DNA binding activity [57]. Taken together, it is likely that activation of the JNK pathway leads to decreased PDX-1 activity and consequent suppression of insulin gene transcription found in the diabetic state.

Also, it was shown that PDX-1 is translocated from the nuclei to the cytoplasm in response to oxidative stress. When β-cell-derived HIT cells were exposed to oxidative stress, PDX-1 moved from the nuclei to the cytoplasm [58]. DN-JNK overexpression inhibited the oxidative stress-induced PDX-1 translocation, suggesting that activation of the JNK pathway is involved in PDX-1 translocation by oxidative stress. Furthermore, leptomycin B, an inhibitor, which specifically suppresses the classical, leucine-rich nuclear export signal (NES), inhibited nucleo-cytoplasmic translocation of PDX-1 induced by oxidative stress [58]. Taken together, it is likely that oxidative stress induces nucleo-cytoplasmic translocation of PDX-1 through activation of the JNK pathway, which leads to reduction of its DNA binding activity and suppression of insulin biosynthesis.

**Figure 3:**
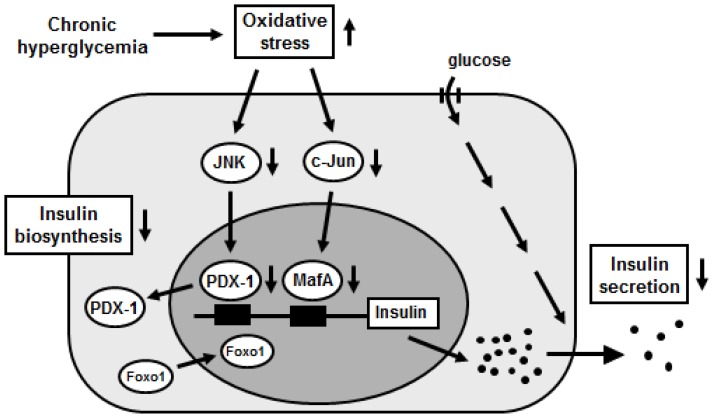
Possible molecular mechanism for suppression of insulin biosynthesis in Type 2 diabetes. Oxidative stress and subsequent activation of the JNK pathway translocate Foxo1 from cytoplasm to nuclei, leading to translocation of PDX-1 from nuclei to cytoplasm in pancreatic β-cells. In addition, oxidative stress and subsequent induction of c-Jun expression suppress nuclear expression of MafA in β-cells. Therefore, it is likely that activation of the JNK pathway and induction of c-Jun expression are involved in suppression of insulin biosynthesis found in Type 2 diabetes.

The forkhead transcription factor Foxo1 is known as one of the important fundamental transcription factors playing a key role in apoptosis, cellular proliferation and differentiation, and glucose metabolism through regulating the transcription of various target genes [59,60]. It was shown that Foxo1 regulates hepatic gluconeogenesis and thus contributes to insulin resistance [61]. Insulin inhibits the function of Foxo1 through Akt-mediated phosphorylation and nuclear exclusion [62], and thereby suppresses hepatic gluconeogenesis. It was also shown that PDX-1 exhibits a counter localization to Foxo1 in β-cells; when Foxo1 is expressed in the cytoplasm, PDX-1 is expressed in the nuclei, and when Foxo1 is expressed in the nuclei, PDX-1 is expressed in the cytoplasm [63]. Moreover, it was shown that Foxo1 plays a role as a mediator between the JNK pathway and PDX-1 [64]. In β-cell-derived cell line HIT cells, Foxo1 changed its intracellular localization from the cytoplasm to the nucleus after exposure to oxidative stress. In contrast to Foxo1, the nuclear expression of PDX-1 was decreased and its cytoplasmic distribution was increased by oxidative stress. Activation of the JNK pathway also induced the nuclear localization of Foxo1. In addition, oxidative stress or activation of the JNK pathway decreased Akt phosphorylation in HIT cells, leading to the decreased phosphorylation of Foxo1 following nuclear localization [64]. Taken together, oxidative stress and subsequent activation of the JNK pathway induce nuclear translocation of Foxo1 through the modification of the insulin signaling in β-cells, which leads to the nucleo-cytoplasmic translocation of PDX-1 and reduction of its DNA binding activity (Figure 3).

## 4. Induction of c-Jun Expression Is Involved in Pancreatic β-Cell Glucose Toxicity

It is known that c-Jun protein level and activity are increased in response to oxidative stress in various cells. We recently reported that c-Jun expression was not clearly detected in islets of control m/m mice and young diabetic db/db mice, but that the number of c-Jun-positive cells gradually increased with age in the islets of diabetic db/db mice [65]. This expression pattern of c-Jun paralleled the loss of MafA expression. Quantitative real-time PCR analysis using freshly isolated islets from db/db mice clearly showed that the c-Jun mRNA level was significantly increased but that both MafA and insulin mRNA levels were markedly decreased with age [65]. Furthermore, it was recently reported that expression levels of MafA in diabetic patients were lower compared to those in healthy subjects [66]. These results imply that the increased level of c-Jun caused a decrease in MafA and insulin gene expression in old diabetic mice. Furthermore, in db/db mice nuclear MafA expression in pancreatic islets was markedly decreased with age and was not clearly detected in old mice, whereas in control m/m mice MafA expression was retained up to old age [65]. In db/db mice insulin expression was also decreased in some cells in which MafA was undetectable or weakly expressed. MafA and insulin expression was suppressed in most c-Jun-positive cells. Similarly, in obese diabetic KK-Ay islets, the number of c-Jun-positive cells was increased with marked hyperglycemia, and both MafA and insulin protein levels were decreased in those cells [65]. These findings suggest that c-Jun is involved in the suppression of MafA and insulin expression under diabetic conditions. In addition, c-Jun overexpression markedly decreased insulin promoter activity, which was consistent with previous reports [67,68].

Although c-Jun protein expression was almost undetectable in MIN6 cells, adenoviral c-Jun overexpression markedly suppressed MafA protein levels and its DNA-binding activity in MIN6 cells [65]. Adenoviral overexpression of c-Jun in isolated mouse islets also markedly suppressed MafA mRNA and protein levels. Consistent with these results, mRNA levels of insulin 1 and 2 and insulin content were suppressed by c-Jun overexpression in both MIN6 cells and islets [65]. These findings directly demonstrate that c-Jun suppresses the expression of both MafA and insulin. In addition, since MafA appears to not only regulate insulin expression but also to be involved in insulin secretion [69,70], it is likely that the suppression of MafA protein levels by c-Jun leads to insulin secretory defects that are often observed under diabetic conditions. In conclusion, the augmented expression of c-Jun in diabetic islets decreases MafA activity followed by reduced insulin biosynthesis and secretion, and thereby explains, at least in part, the molecular mechanism for β-cell glucose toxicity that is often observed in Type 2 diabetes (Figure 3).

## 5. Conclusions

Pancreatic β-cell dysfunction and insulin resistance are the hallmarks of Type 2 diabetes. Under diabetic conditions, hyperglycemia *per se* and subsequent induction of oxidative stress decrease insulin biosynthesis and secretion, accompanied by reduction in expression and/or activities of insulin gene transcription factors PDX-1 and MafA. This process is known as β-cell glucose toxicity, which is often observed under diabetic conditions.
